# The Precipitation of Gold

**Published:** 1885-02

**Authors:** 


### The Precipitation of Gold.

If we compare the various processes for the precipitation of gold, it appears that the method with ferrous sulphate in an acid solution is simple in execution and complete, provided only the solution is free from chlorine, bromine, nitric acid, and from calcium, magnesium, and sodium hypochlorites. This is not the case with the mother liquors of chlorination processes, which may contain all the above mentioned bodies. Ferrous chloride has the same effect, but is dear, easily decomposed, and can be conveyed only in vessels of glass or porcelain. The precipitation with hydrogen is complicated, as a special apparatus and a temperature of 50° to 60° are requisite. It is, however, applicable in all cases, if no copper is present in the solution. The precipitate settles quickly after the reduction of all the oxidized compounds.—*Chemiker Zeitung, Goethen.*

